# High serum levels of the C-propetide of type V collagen (PRO-C5) are prognostic for short overall survival in patients with pancreatic ductal adenocarcinoma

**DOI:** 10.3389/fmolb.2023.1158058

**Published:** 2023-03-10

**Authors:** Neel I. Nissen, Astrid Z. Johansen, Inna M. Chen, Christina Jensen, Emilie A. Madsen, Carsten P. Hansen, Jeppe Thorlacius-Ussing, Morten Karsdal, Julia S. Johansen, Hadi M. H. Diab, Lars N. Jørgensen, Nicholas Willumsen

**Affiliations:** ^1^ Nordic Bioscience A/S, Herlev, Denmark; ^2^ Department of Oncology, Copenhagen University Hospital, Gentofte, Denmark; ^3^ Department of Surgery, Copenhagen University Hospital – Rigshospitalet, Copenhagen, Denmark; ^4^ Institute of Clinical Medicine, Faculty of Health and Medical Sciences, University of Copenhagen, Copenhagen, Denmark; ^5^ Department of Medicine, Copenhagen University Hospital, Gentofte, Denmark; ^6^ Digestive Disease Center, Bispebjerg and Frederiksberg Hospital, University of Copenhagen, Copenhagen, Denmark

**Keywords:** cancer, collagen, PDAC, prediction, prognosis, tumor fibrosis, type V collagen

## Abstract

**Introduction:** Pancreatic ductal adenocarcinoma (PDAC) is characterized by a pronounced fibrotic tumor microenvironment, which impairs treatment response. Type I and V collagens are responsible for the densely packed fibrils in the tumor fibrosis environment. While the role of the major type I collagen in cancer is well described, less is known about the minor type V collagen. Quantifying collagen propeptides in serum has been shown to have prognostic and predictive value. In this study, we evaluated the clinical utility of measuring the propeptide of type V collagen (PRO-C5) in serum from a discovery cohort and a validation cohort of patients with PDAC as well as in non-pancreatic solid tumor types to explore the relevance of the PRO-C5 biomarker in cancer.

**Methods:** Serum PRO-C5 was measured in three cohorts: a discovery cohort (19 healthy controls, 12 patients with chronic pancreatitis and 33 patients with PDAC (stage I-IV)), a validation cohort (800 patients with PDAC (stage I-IV)), and a non-pancreatic solid tumor type cohort of 33 healthy controls and 200 patients with 10 different non-pancreatic solid tumor types. The levels of serum PRO-C5 in patients with cancer were compared to levels in healthy controls. The association between PRO-C5 levels and overall survival (OS) was evaluated in patients with PDAC after adjusting for established prognostic factors.

**Results:** PRO-C5 was significantly increased in serum from patients with PDAC compared to healthy controls (*p* < 0.001). High PRO-C5 levels were significantly associated with short OS in both the discovery- and the validation cohort, especially in early stages of PDAC (validation cohort stage II, HR = 2.0, 95%CI1.2-3.4). The association was independent of other prognostic parameters including stage, performance status and CA19-9. Furthermore, serum levels of PRO-C5 were significantly increased in serum from patients with other non-pancreatic solid tumor types compared to healthy controls.

**Conclusion:** High levels of serum PRO-C5 is prognostic for short OS in patients with PDAC and may provide clinical value in many other tumor types beyond PDAC. This underlines the importance of type V collagen in tumor fibrosis. PRO-C5 could have the potential to be used in several aspects within drug discovery, patient stratification and drug efficacy.

## 1 Introduction

Pancreatic ductal adenocarcinoma (PDAC) is a devastating disease as only 20% of the patients have resectable disease (McGuigan et al., 2018; Klein, 2019; Khalaf et al., 2021; Siegel et al., 2021). The remaining patients can only be offered palliative oncologic treatment or best supportive care (McGuigan et al., 2018). However, there is a high degree of treatment resistance to oncologic treatment. One explanation is an extremely fibrotic PDAC tumor microenvironment. Tumor fibrosis cause increased interstitial pressure, which in turn reduces drug delivery (Heldin et al., 2004; Thomas and Radhakrishnan, 2019). In addition, tumor fibrosis inhibits T-cell activity and migration, which results in diminished efficacy of immunotherapy (Mariathasan et al., 2018; Chen et al., 2021; Ogawa et al., 2021; Grout et al., 2022; Lander et al., 2022). Therefore, there is a high need for novel strategies to overcome tumor fibrosis in the treatment of patients with PDAC and consequently to identify tumors with high fibrotic activity.

Tumor fibrosis is characterized by an augmented activity of cancer-associated fibroblasts (CAFs), responsible for an abnormal extracellular matrix (ECM) remodeling (Nissen et al., 2019). The CAF-mediated ECM remodeling results in degradation of existing collagen fibers that are replaced by new and more densely packed collagen fibers, creating a stromal barrier surrounding the tumor cells (Nissen et al., 2019) (Winkler et al., 2020). Type I collagen is the most abundant CAF-derived fibrillar collagen and a major component of the collagen fibers that play important roles during tumor progression (Menke et al., 2001; Armstrong et al., 2004; Cheng and Leung, 2011; Gao et al., 2016; Barcus et al., 2017; Chen et al., 2022). The pathogenic phenotype of CAFs was originally thought to derive from the ability to produce large quantities of collagen, but recent studies focusing on type I collagen have emphasized the importance of the collagen quality and fiber architecture, as a central component in its tumorigenic capacity (Chen et al., 2022; Su et al., 2022). Interestingly, the minor fibrillar type V collagen has been suggested to be a key regulator of type I collagen architecture.

**FIGURE 1:**
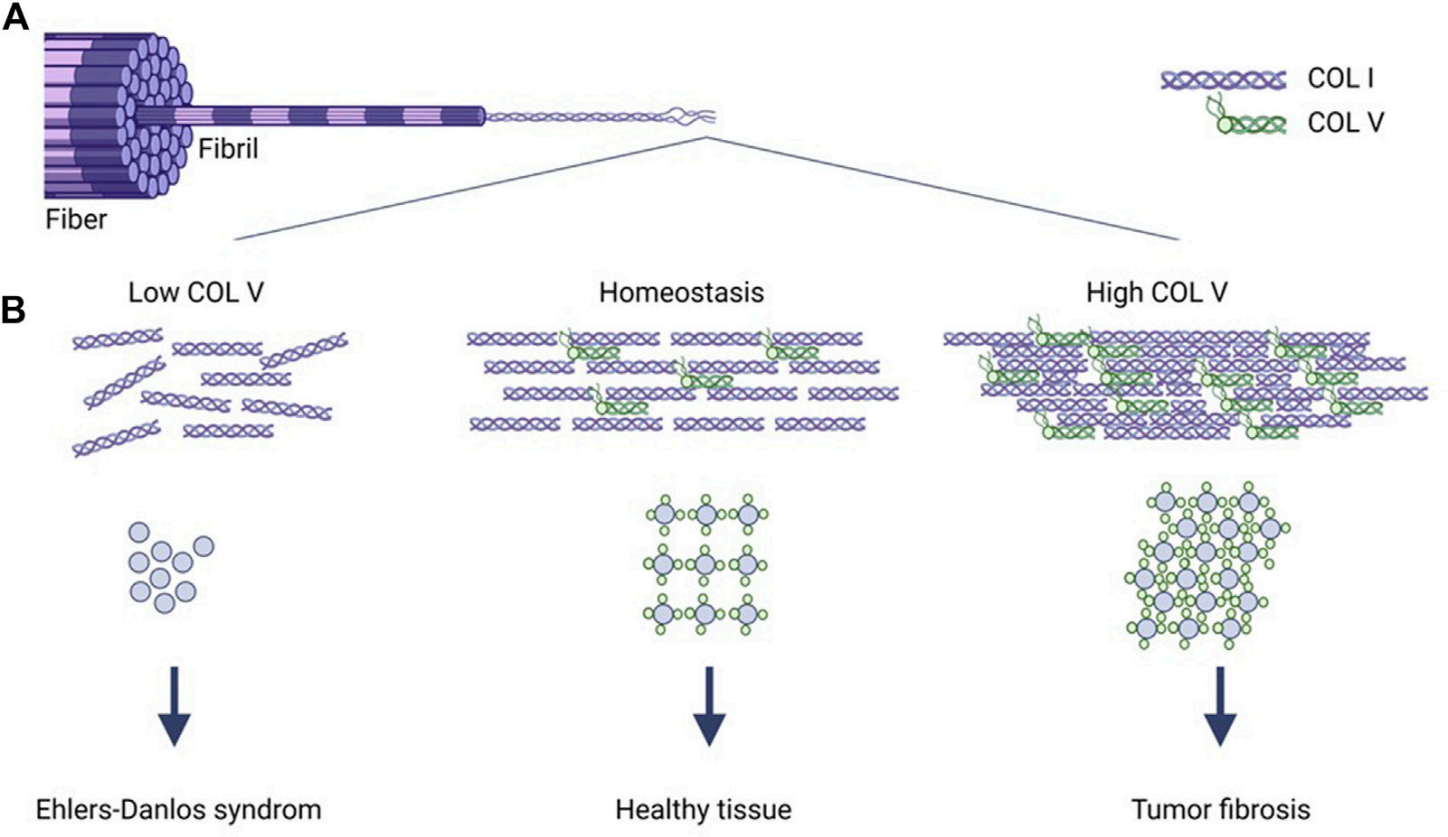
Type V collagen regulates type I collagen architecture. Collagen fibers consist of collagen fibrils, which are made up by self-assembled collagen microfibrils **(A)**. Type V collagen is incorporated into the type I collagen microfibril structure, where it during homeostasis regulates structural integrity of type I collagen **(B)**. Type V collagen (low COLV) deficiency results in abnormal type I collagen fibrils and the well-known Ehlers-Danlos syndrome **(B)**. Augmented type V collagen expression (high COLV) results in more condensed and linearized type I collagen fibrils and dense fibers characteristic of tumor fibrosis **(B)**. COLI, type I collagen; COLV, type V collagen.

Type V collagen has many roles in the healthy ECM, as it binds to different ECM proteins such as other collagens, TGF-β, elastin and metalloproteinases, thereby modulating cellular behavior (Symoens et al., 2010). The most abundant isoform of type V collagen consist of two α1 chains and one α2 chain that forms a heterotrimer. In healthy tissue, type I and V collagen have a very close relationship, as the two collagens copolymerize into heterotypic fibrils (Wenstrup et al., 2004; Aszódi et al., 2006; Sun et al., 2011) (Figure 1). The binding of type V collagen to type I collagen is important for structural integrity and deficiency of type V collagen results in disorders like Ehlers-Danlos syndrome and corneal disease, which are characterized by abnormal collagen fibrils (Mak et al., 2016). Type V collagen deficient mice (COL5a1^−/−^) embryos die already at day 10.5 as they also lack type I collagen fibrils. Moreover, heterozygous type V collagen mice (COL5a1^−/+^) survive, but show 50% decrease in collagen content and fibrillar density (Wenstrup et al., 2004). Thus, the binding between type V and I collagen results in a homeostatic fibril structure and tissue architecture. However, a complete understanding of its role in tumor progression remains (Mak et al., 2016). In comparison to type I collagen, type V collagen is expressed in relatively low levels in healthy tissues. In cancer, type V collagen, especially the α2 chain, has been shown to be upregulated in colorectal, gastric and breast cancer and is associated with PDAC cell proliferation, invasion, metastasis, and angiogenesis (Barsky et al., 1982; Imamura et al., 1995; Sun et al., 2011; Berchtold et al., 2015; Mak et al., 2016; Huang et al., 2017; Ren et al., 2018; Tan and Chen, 2018; Balancin et al., 2020; Wang et al., 2021a; Tan et al., 2021). Altogether, this indicates, that type V collagen may be a key factor in determining fibrillar density and hence tumor fibrosis.

The present study explored the biomarker potential of measuring the C-propeptide of the α2 chain of type V collagen (PRO-C5) in serum from a discovery cohort and a validation cohort of patients with PDAC as well as in 10 non-pancreatic solid tumor types to explore if the PRO-C5 biomarker had relevance in PDAC and other tumor types.

## 2 Materials and methods

### 2.1 Patients

The PDAC discovery cohort (Cohort 1), consisted of 19 age- and sex-matched healthy controls, 12 patients with chronic pancreatitis and 33 patients with PDAC (stage I-IV). The PDAC validation cohort (Cohort 2), consisted of 800 patients with PDAC (stage I-IV). Serum samples from patients with chronic pancreatitis and PDAC in cohort 1 and 2 were from the Danish BIOPAC study “Biomarkers in patients with pancreatic cancer (BIOPAC) – can they provide new information of the disease and improve diagnosis and prognosis of the patients” (ClinicalTrials.gov ID: NCT03311776). The study has been described elsewhere (Chen et al., 2019; Bagni et al., 2020; Nissen et al., 2021a; Nissen et al., 2022). The study was carried out in accordance with the Danish Regional Committee on Health Research Ethics. The BIOPAC protocol was approved by the Danish Regional Committee on Health Research Ethics (VEK ref. KA-20060113; and the retrospective protocol VEK H-17039022) and the Data Protection Agency (j.nr. 2006-41-6848, 2012-58-0004, HGH-2015-027; I-Suite j. nr. 03960; and PACTIUS P-2020-834). All subjects gave written informed consent in accordance with the Declaration of Helsinki, version 8. Serum samples were collected before operation or before start of palliative chemotherapy. Clinical data were collected prospectively. The patients were followed until September 2022 or death, whichever came first. Serum samples were measured blinded without information of the clinical characteristics. Clinical data included age, sex, stage (American Joint Commission on Cancer, eighth edition), number of metastatic sites, liver metastasis, body mass index (BMI), diabetes, tobacco use, alcohol use, carbohydrate antigen 19-9 (CA19-9), performance status (PS), Charlson age comorbidity index (CACI) and overall survival (OS). Cohort 3 consisted of 33 age and sex matched healthy controls and 200 patients with different types of non-pancreatic cancers: bladder (n = 20), breast (n = 20), colorectal (n = 20), head and neck (n = 20), kidney (n = 20), lung (n = 20), malignant melanoma (n = 20), ovarian (n = 20), prostate (n = 20), and stomach (n = 20). Serum from patients with cancer in cohort 3 were obtained from the commercial vendor Proteogenex (CA, United States). Healthy controls in cohort 1 and 3 were obtained from Valley BioMedical (VA, United States) and BioIVT (Westbury, NY, United States), respectively. Appropriate Institutional Review Board/Independent Ethical Committee approved sample collection and all subjects filed for informed consent. Patient demographics for cohort 1, 2 and 3 are shown in Tables 1, 2, respectively.

**TABLE 1:** Patient demographics for Cohort 1 and Cohort 2.

Discovery cohort (cohort 1)		Validation cohort (cohort 2)
**Healthy controls**	**n = 19**	
Age, (years)		
*Median* (*min, max*)	58 (45-72)	
Gender, n (%)		
*Male*	10 (53%)	
*Female*	9 (47%)	
**Chronic pancreatitis**	**n = 12**	
Age, (years)		
*Median* (*min, max*)	60 (49-79)	
Gender, n (%)		
*Male*	10 (83%)	
*Female*	2 (17%)	
**PDAC**	**n = 33**	**n = 800**
Age, (years)		
*Median* (*min, max*)	69 (52-79)	67 (37-89)
Gender, n (%)		
*Male*	17 (52%)	428 (54%)
*Female*	16 (48%)	372 (46%)
Number of metastatic sites, n (%)		
*0 site*	15 (45%)	364 (45%)
*≥1 site*	18 (55%)	436 (55%)
*Liver metastasis* (*of all patients with metastasis, n = 18*)*, n* (*%*)		*Liver metastasis (of all patients with metastasis, n = 436), n (%)*
*Yes*	17 (94%)	324 (74%)
*No*	1 (6%)	112 (26%)
BMI (kg/m^2^)		
*Median* (*min, max*)	23 (16-31)	23 (14-39)
Stage		
*1*	1 (3%)	15 (2%)
*2*	7 (21%)	119 (15%)
*3*	7 (21%)	227 (28%)
*4*	18 (55%)	435 (54%)
*Unknown*	0	4 (<1%)
Diabetes		
*Yes*	8 (24%)	194 (24%)
*No*	25 (76%)	597 (75%)
*Unknown*	0	9 (1%)
Tobacco		
*Ever*	21 (64%)	485 (61%)
*Never*	12 (36%)	244 (31%)
*Unknown*	0	71 (8%)
Alcohol		
*<DHAR*	26 (79%)	551 (69%)
*>DHAR*	7 (21%)	176 (22%)
*Unknown*	0	73 (9%)
CA19-9 (U/mL)		
≤*median 483 U/mL*	15 (45%)	386 (48%)
*>median 483 U/mL*	18 (55%)	388 (49%)
*Unknown*	0	26 (3%)
Performance status, n (%)		
*0*	13 (39%)	286 (36%)
*1*	12 (36%)	335 (42%)
*2*	3 (9%)	90 (11%)
*3*	0	4 (<1%)
*Unknown*	5 (15%)	85 (11%)
Charlson age comorbidity index		
*<4*	n/a	679 (85%)
*≥4*	n/a	108 (14%)
*Unknown*	n/a	13 (1%)

Abbreviations: BMI, body mass index; CA 19-9, carbohydrate antigen 19-9; DHAR, danish health authority recommendations.

**TABLE 2:** Patient demographics for Cohort 3.

	Healthy controls (n = 33)	Patients with cancer (n = 200)
**Age (years)**		
Median (min; max)	57.0 (49.0-69.0)	61.0 (30.0-87.0)
**Sex**		
Male	21 (64%)	119 (54%)
Female	12 (36%)	101 (46%)
**Cancer type**		
Bladder		20 (10%)
Breast		20 (10%)
Colorectal		20 (10%)
Head & neck		20 (10%)
Renal		20 (10%)
Lung		20 (10%)
Melanoma		20 (10%)
Ovarian		20 (10%)
Prostate		20 (10%)
Gastric		20 (10%)
**Stage**		
I		7 (3%)
II		46 (21%)
III		93 (42%)
IV		74 (34%)

### 2.2 Assessment of the C-propeptide of type V collagen fragments (PRO-C5) in human serum

Serum levels of the C-propeptide of type V collagen were measured using the ELISA based assay PRO-C5 according to manufacturer´s instruction (Nordic Bioscience A/S, Denmark). The technical details of the assays have previously been described (Vassiliadis et al., 2011; Vassiliadis et al., 2012).

### 2.3 Statistics

Biomarker results were reported in accordance with the REMARK (reporting recommendations for tumor marker prognostic study) guidelines (Altman et al., 2012). A Kruskal–Wallis multiple comparison test was used to test the difference between PRO-C5 serum levels in healthy controls, and patients with either chronic pancreatitis or PDAC (cohort 1) as well as for PRO-C5 serum levels in healthy controls and in 10 different cancer indications (cohort 3). Non-parametric Mann Whitney tests were used to assess associations with PRO-C5 serum levels in early (stage I-II) and late (stage III-IV) stages of PDAC in cohort 1 and cohort 2. Kaplan-Meier curves were used to assess the association between high and low serum levels of PRO-C5 and OS. To provide granularity on the association between PRO-C5 and overall survival, and to explore a potential cut off proximity to be tested in the validation cohort, the patients in the discovery cohort, cohort 1, were stratified into four groups based on quartiles (Q1, Q2, Q3 and Q4). In cohort 2, patients were stratified based on the 75th percentile, meaning Q1-Q3 vs. Q4 (cut-off: 832 ng/ml). When exploring the association between PRO-C5 serum levels and OS in specific stages of PDAC, patients were stratified based on the 75th percentile, meaning Q1-Q3 vs. Q4 (Stage II cut-off 725 ng/ml; stage III cut-off 792 ng/ml; stage IV cut-off 841 ng/ml). In cohort 1, a univariate Cox proportional-hazard regression model was used to calculate the hazard ratios (HR) with 95% confidence interval (Cl) for short OS per PRO-C5 biomarker levels (Q4, or Q3 or Q2 vs. Q1). In cohort 2, a univariate Cox proportional-hazard regression model was used to calculate the hazard ratios (HR) with 95% confidence interval (Cl) for short OS per PRO-C5 biomarker levels (continuous and Q1-Q3 vs. Q4) and clinical covariates: age (continuous), gender (female vs. male), number of metastatic sites (≥1 vs. 0), liver metastasis (yes vs. no), BMI (continuous), stage (I-II vs. III-IV), diabetes (yes vs. no), tobacco use (ever vs. never), alcohol use (below and above the Danish Health Authority recommendations [DHAR]), CA19-9 (>median vs. ≤ median [median = 483 U/mL]), PS (1 + 2 + 3 vs. 0) and CACI (≥4 vs. < 4). In addition, in cohort 2, a multivariate Cox proportional-hazard regression models including PRO-C5 (continuous and Q1-Q3 vs. Q4), age, metastatic sites (≥1 vs. 0), liver metastasis (yes vs. no), stage (I-II vs. III-IV), CA19-9 (>median vs. ≤ median [median = 483 U/mL]), PS (1 + 2 + 3 vs. 0) and CACI (≥4 vs. < 4) was used to evaluate potential independent prognostic value of the PRO-C5 biomarker for predicting OS. When the model was used for patients in individual stages only age, CA19-9 (>median vs. ≤ median [median = 483 U/mL]), PS (1 + 2 + 3 vs. 0) and CACI (≥4 vs. < 4) were included for stage II and III. For stage IV, age, CA19-9 (>median vs. ≤ median [median = 483 U/mL]), PS (1 + 2 + 3 vs. 0), CACI (≥4 vs. < 4), number of metastatic sites (≥1 vs. 0) and liver metastasis (yes vs. no) were included. Patients in disease stage I were not analyzed due to the low number of patients in this group (n = 15). A *p*-value of *p* < 0.05 was considered statistically significant. Graph design and statistical analyses were performed using GraphPad Prism Version 9 (GraphPad Software, Inc.) and MedCalc version 19.3 (Medcalc Software).

## 3 Results

### 3.1 PRO-C5 is elevated in patients with PDAC and associates with poor OS–Discovery cohort

**FIGURE 2:**
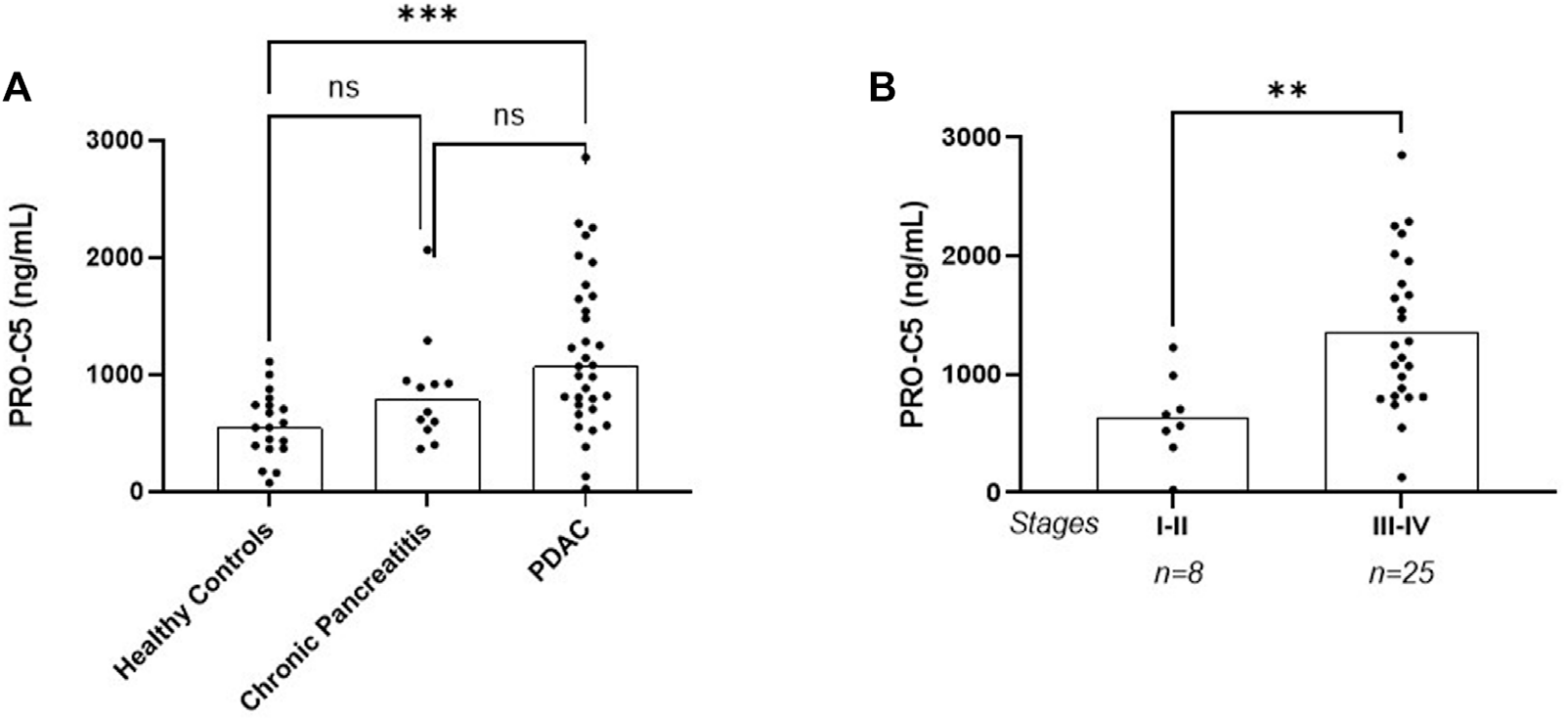
PRO-C5 is elevated in serum from patients with pancreatic ductal adenocarcinoma (PDAC) in the discovery cohort. **(A)** Individual serum PRO-C5 levels in patients with PDAC (n = 33), chronic pancreatitis (n = 12) and healthy controls (19). Differences in PRO-C5 levels between disease indication were analyzed with a Kruskal–Wallis multiple comparison test non-parametric test. **(B)** Individual serum PRO-C5 levels in patients with early (stage I-II) and late (stage III-IV) stage PDAC. Differences in PRO-C5 levels between early and late stage PDAC were analyzed with a non-parametric Mann Whitney test. Ns, *p* > 0.05; **p < 0.01; ***p < 0.001.

In the discovery cohort, cohort 1, PRO-C5 was significantly elevated in serum from patients with PDAC compared to healthy controls (median PRO-C5 values: PDAC 1071 ng/ml vs. healthy controls 550 ng/ml, *p* < 0.001). PRO-C5 was not significantly increased in patients with chronic pancreatitis (median PRO-C5 value 786 ng/ml) compared to healthy controls nor in patients with PDAC compared to chronic pancreatitis (Figure 2A). When stratifying patients with PDAC into early and late stage (stage I-II vs. III-IV), patients in late stage PDAC had significantly increased PRO-C5 (median PRO-C5 values: 635 ng/ml vs. 1360 ng/ml, respectively, *p* = 0.0018) (Figure 2B).

**FIGURE 3:**
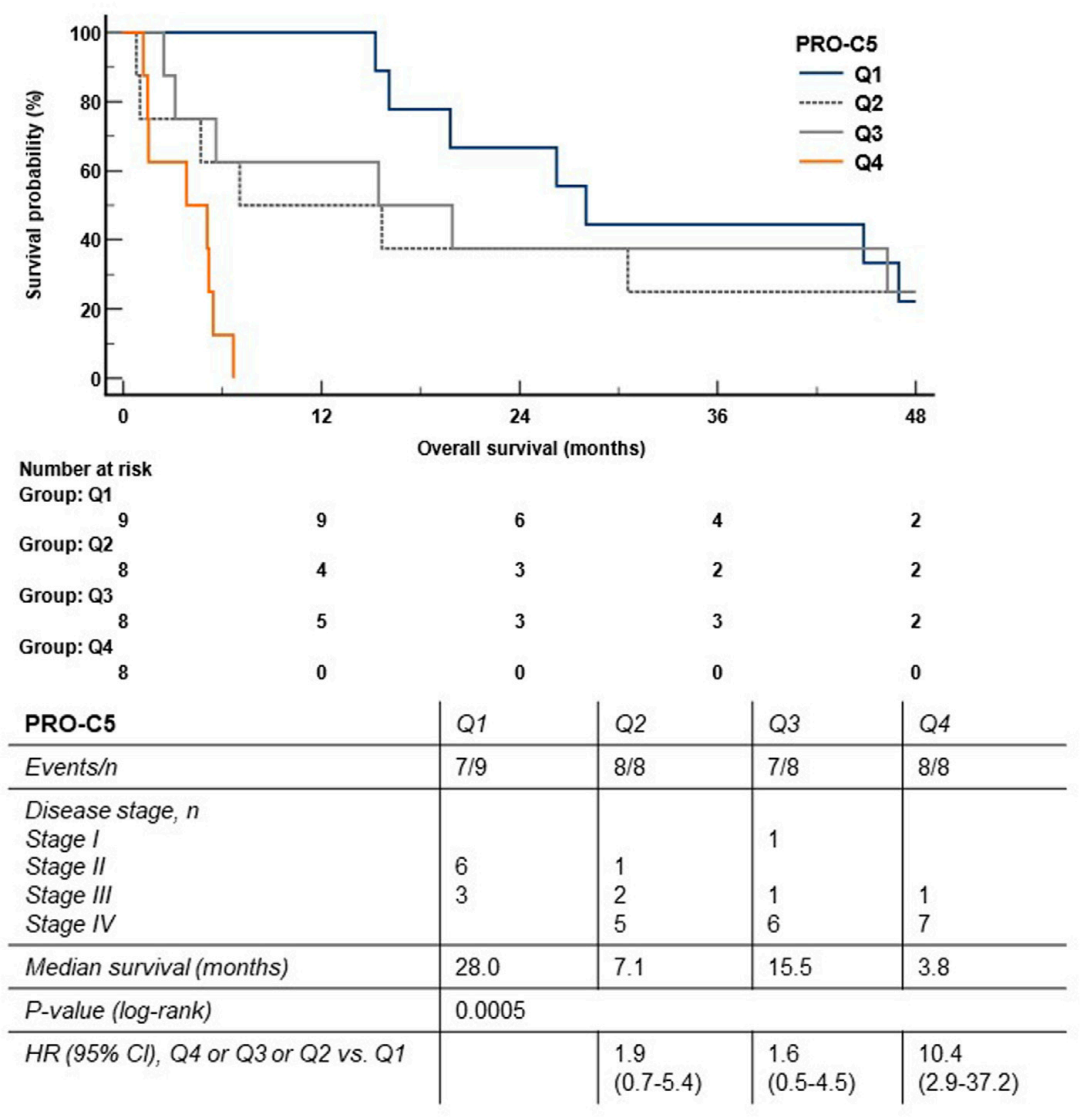
High levels of serum PRO-C5 were associated with short overall survival in the discovery cohort. The Kaplan-Meier survival plot shows the association between overall survival and serum PRO-C5. Patients were stratified into quartiles (Q1 (blue) patient with the lowest PRO-C5; and Q4 (orange) patients with the highest levels of PRO-C5). Hazard ratios (HR), 95% confidence intervals (CI) and log-rank test are shown.

To investigate the association between PRO-C5 serum levels and OS, we assessed the prognostic potential of PRO-C5 by Kaplan-Meier curves and a univariate Cox proportional-hazard model. Patients were stratified into quartiles i.e., Q1 containing patient with the lowest levels of PRO-C5 and Q4 containing patients with the highest levels of PRO-C5. Patients in Q1, Q2, Q3 and Q4 had median OS of 28.0 months, 7.1 months, 15.5 months, and 3.8 months, respectively (log-rank, *p* = 0.0005). Thus, the difference in median OS between Q4 and Q1 were more than 2 years (24.2 months) (Figure 3). In support, univariate Cox proportional-hazard model showed that patients in Q4 had a 940% increased risk of mortality compared with patients in Q1 (Q4 vs. Q1: HR = 10.4, 95% CI 2.9-37.2, *p* = 0.0003) (Figure 3). There was no significant difference in risk of mortality and PRO-C5 levels between Q4 vs. Q2 and Q4 vs. Q3 (Figure 3).

### 3.2 PRO-C5 is elevated in patients with PDAC and associates with poor OS–Validation cohort

**FIGURE 4:**
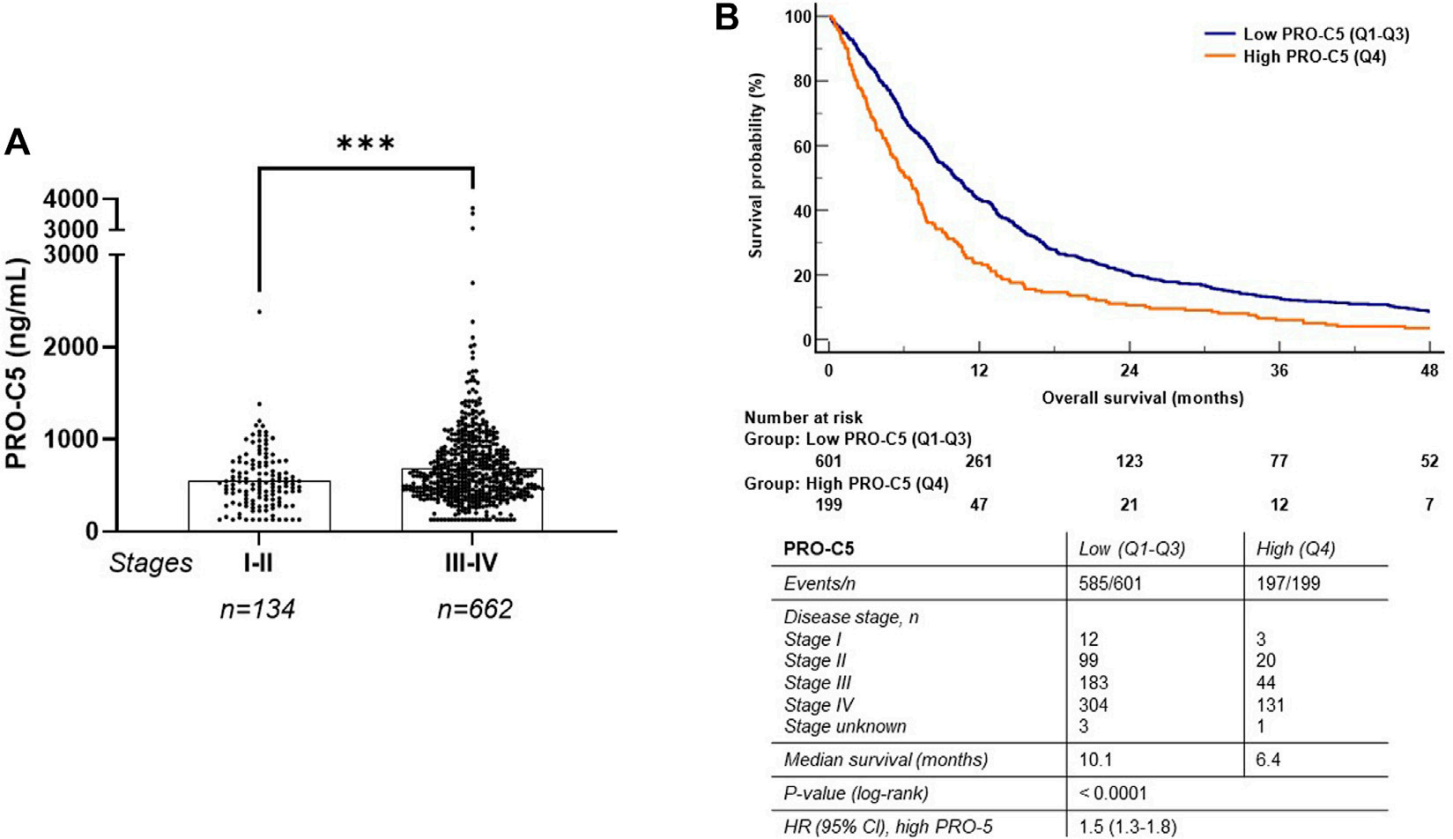
High levels of serum PRO-C5 were associated with short overall survival in the validation cohort. **(A)** Individual serum PRO-C5 levels in patients with early (stage I-II) and late (stage III-IV) PDAC. Differences in PRO-C5 levels between early and late stage PDAC were analyzed with a non-parametric Mann Whitney test. **(B)** A Kaplan-Meier survival plot shows the association between overall survival and levels of PRO-C5. Patients were stratified into low (Q1-Q3, blue) and high (Q4, orange) PRO-C5 (cutoff, 832 ng/ml). Hazard ratios (HR), 95% confidence intervals (CI) and log-rank test are shown. ***p < 0.001.

In the validation cohort, cohort 2, we were overall able to confirm the findings from the discovery cohort including a PRO-C5 increase in late stage PDAC compared to early stage PDAC (*p* = 0.004) (Figure 4A). To evaluate the association between PRO-C5 serum levels and OS in the PDAC validation cohort, patients were stratified into two groups based on the quartiles similar to the approach used in cohort 1 above: one containing patients with low levels of serum PRO-C5 (below the Q4 percentile), and another containing patients with high levels of serum PRO-C5 (above the Q4 percentile). Patients with low and high PRO-C5 had a median OS of 10.1 and 6.4 months, respectively (log-rank *p* < 0.0001) (Figure 4B). In addition, univariate Cox proportional-hazard modelling showed that patients with high levels of PRO-C5 had a 50% higher risk of mortality as compared to patients with low levels of PRO-C5 (High PRO-C5 vs. low PRO-C5: HR = 1.5, 95% CI 1.3-1.8, *p* < 0.0001) (Figure 4B and Table 3). To evaluate if the association of OS and PRO-C5 was independent of clinical covariates, a multivariate Cox proportional-hazard model including age, number of metastatic sites, liver metastasis, stage, CA19-9, PS and CACI was performed. The model showed that the prognostic value of PRO-C5 remained statistically significant when adjusting for these clinical covariates (HR = 1.4, 95% CI 1.2-1.7, *p* = 0.0001) (Table 3), which indicated that PRO-C5 is a risk factor independent of other common risk factors.

**TABLE 3:** Uni- and multivariate cox regression analysis in 800 patients with pancreatic ductal adenocarcinoma stage I-IV.

Cox regression analysis		Univariate		Multivariate	
Variables		HR (95% CI)	*p*-value	HR (95% CI)	*p*-value
**PRO-C5**	Continuous	1.0 (1.0-1.0)	<0.0001	-	-
**PRO-C5**	Q4 vs. Q1-Q3	1.5 (1.3-1.8)	<0.0001	1.4 (1.2-1.7)	0.0001
**Age per year**	Continuous	1.0 (1.0-1.0)	0.0356	1.0 (1.0-1.0)	0.2646
**Sex**	Female vs. male	1.0 (0.8-1.1)	0.6569	-	-
**PDAC stage**	I-II vs. III-IV	2.9 (2.4-3.5)	<0.0001	2.0 (1.6-2.5)	<0.0001
**No. of metastatic sites**	≥1 vs. 0	2.6 (2.5-3.0)	<0.0001	1.6 (1.3-2.0)	<0.0001
**Liver metastasis**	Yes vs. No	2.3 (2.0-2.7)	<0.0001	1.1 (0.9-1.4)	0.2401
**BMI per kg/m** ^**2**^	Continuous	1.0 (1.0-1.0)	0.2715	-	-
**Diabetes**	Yes vs. no	1.1 (0.9-1.3)	0.3616	-	-
**Alcohol intake**	>DHAR vs. <DHAR	1.0 (0.8-1.3)	0.8814	-	-
**CA19-9**	>median vs. <median	2.0 (1.7-2.3)	<0.0001	1.5 (1.3-1.8)	<0.0001
**PS**	1 + 2+3 vs. 0	1.5 (1.3-1.8)	<0.0001	1.6 (1.3-1.8)	<0.0001
**CACI**	High (≥4 vs. <4)	1.3 (1.0-1.5)	0.0315	1.2 (0.9-1.4)	0.2140

Abbreviations: BMI, body mass index; CACI, charlson age comorbidity index; CA 19-9, carbohydrate antigen 19-9; DHAR, Danish Health Authority recommendations; No, number; PDAC, pancreatic ductal adenocarcinoma; PS, performance status; HR, hazard ratios; and CI, confidence intervals.

Next, we wanted to gain more granularity on the association between high serum PRO-C5 and OS in patients stratified into specific stages of PDAC. Patients in disease stage I were not analyzed due to the low number of patients in this group (n = 15). For each cancer stage, patients were stratified into high and low PRO-C5 using the 75th percentile as a cutoff.

**FIGURE 5:**
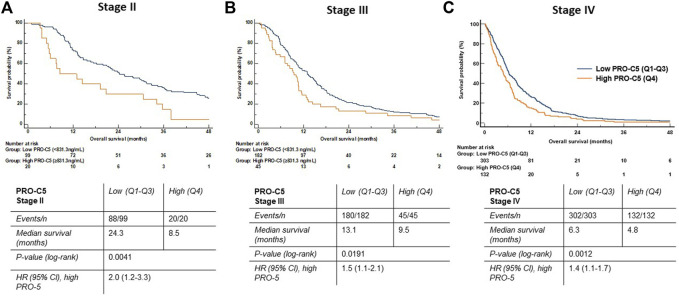
High levels of serum PRO-C5 were associated with short overall survival in patients with PDAC according to stage II, III and IV in the validation cohort. Kaplan-Meier survival plots show the association between overall survival and levels of PRO-C5 in stage II **(A)**, stage III **(B)** and stage IV patients **(C)**. In all plots, patients were stratified into low (Q1-Q3, blue) and high (Q4, orange) PRO-C5 (Stage II: cut-off 725 ng/ml, stage III: cut-off 792 ng/ml, stage IV: cut-off 841 ng/ml). Hazard ratios (HR), 95% confidence intervals (CI) and log-rank test are shown.

Patients with stage II disease (n = 117) and low levels of PRO-C5 had a medium OS at 24.3 months compared to 8.5 months in patients with low PRO-C5 (log rank test, *p* = 0.0041) (Figure 5A). Thus, the difference in median OS between the two groups was more than 1 year (15.8 months). Univariate Cox proportional-hazard model showed that patients with stage II PDAC and high levels of PRO-C5 had a 100% increased risk of death compared to patients with low PRO-C5 (HR = 2.0, 95% CI 1.2-3.3, *p* = 0.0049) (Figure 5A). A multivariate Cox proportional-hazard model including age, CA19-9, PS and CACI showed that the association between high PRO-C5 and short OS was independent of the clinical variables in patients with stage II disease (HR = 2.0, 95% CI 1.2-3.4, *p* = 0.010) (Table 4).

**TABLE 4:** Multivariate Cox proportional-hazard regression model in patients with pancreatic ductal adenocarcinoma divided into stage II, III and IV.

		Stage II		Stage III		Stage IV	
		N = 117		N = 227		N = 435	
Variables		HR (95% CI)	*p*-value	HR (95% CI)	*p*-value	HR (95% CI)	*p*-value
**PRO-C5**	Q4 vs. Q1-Q3	2.0 (1.2-3.4)	0.0100	1.5 (1.1-2.1)	0.0177	1.3 (1.1-1.6)	0.0085
**Age**	Continuous	1.00 (1.0-1.0)	0.3620	1.0 (1.0-.10)	0.1825	1.0 (1.0-1.0)	0.5004
**CA19-9**	>median vs. <median	1.3 (0.9-2.0)	0.1711	1.6 (1.2-2.0)	0.0010	1.3 (1.1-1.6)	0.0129
**PS**	1 + 2+3 vs. 0	1.1 (0.7-1.7)	0.7235	1.2 (0.9-1.6)	0.1694	1.6 (1.3-1.9)	<0.0001
**CACI**	High (≥4 vs. <4)	1.2 (0.6-2.2)	0.6227	1.5 (1.0-2.3)	0.0577	1.2 (1.0-1.5)	0.0971
**No. of metastatic sites**	≥1 vs. 0	-	-	-	-	1.0 (0.8-1.3)	0.9871
**Liver metastases**	Yes vs. No	-	-	-	-	1.2 (1.0-1.5)	0.1427

Abbreviations: CACI, charlson age comorbidity index; CA 19-9, carbohydrate antigen 19-9; No, number; PS, performance status; Hazard ratios, HR; CI, confidence intervals.

Patients with stage III disease (n = 227) and low levels of PRO-C5 had a median OS of 13.1 months compared to 9.5 months in patients with high PRO-C5 (log rank test, *p* = 0.0191) (Figure 5B). Univariate Cox proportional-hazard model showed that patients with stage III disease and high PRO-C5 had a 50% increased risk of death compared to patients with low PRO-C5 (HR = 1.5, 95% CI 1.1-2.1, *p* = 0.0191) (Figure 5B). A multivariate Cox proportional-hazard model including age, CA19-9, PS and CACI showed that the association between high PRO-C5 and short OS was independent of the clinical variables in patients with stage III disease (HR = 1.5, 95% 1.1-2.1, *p* = 0.0177) (Table 4).

Patients with stage IV disease (n = 435) and low levels of PRO-C5 had a median OS of 6.3 months compared to 4.8 months in patients with high PRO-C5 (log rank test, *p* = 0.0012) (Figure 5C). Univariate Cox proportional-hazard model showed that patients with stage IV disease and high PRO-C5 had a 40% increased risk of death compared to patients with low PRO-C5 (HR = 1.4, 95% CI 1.1-1.7, *p* = 0.0012) (Figure 5C). A multivariate Cox proportional-hazard model including age, CA19-9, PS, CACI, number of metastatic sites and liver metastasis showed that the association between high PRO-C5 and short OS was independent of the clinical variables in patients with stage IV disease (HR = 1.3, 95% CI 1.1-1.6, *p* = 0.0085) (Table 4).

### 3.3 PRO-C5 is increased in serum from patients with different non-pancreatic types of cancer

**FIGURE 6:**
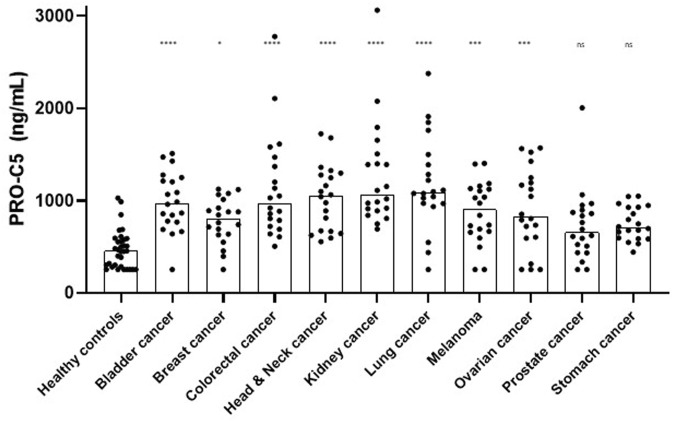
PRO-C5 was elevated in serum from patients with different types of cancer. PRO-C5 individual measurements in patients with different non-pancreatic types of cancer: bladder cancer (n = 20), breast cancer (n = 20), colorectal cancer (n = 20), head and neck cancer (n = 20), kidney cancer (n = 20), lung cancer (n = 20), malignant melanoma (n = 20), ovarian cancer (n = 20), prostate cancer (n = 20) or stomach cancer (n = 20); and compared to healthy controls (n = 33). Bar plots showing the median PRO-C5 value. A Kruskall-Wallis test was used to analyze the difference in PRO-C5 between cancer types. Ns, non-significant; *p < 0.05; ***p < 0.001; ****p < 0.0001.

Next, we wanted to explore the PRO-C5 biomarker potential in other non-pancreatic cancer types (cohort 3). Bladder-, breast-, colorectal-, head and neck-, kidney-, lung-, ovarian cancer and melanoma had significantly elevated PRO-C5 levels compared to healthy controls (*p* < 0.05 to 0.0001), whereas this was not found for patients with prostate- and stomach cancer Figure 6. PRO-C5 levels for individual stages in each cancer disease can be seen in S1.

## 4 Discussion

In this study we evaluated the biomarker potential of measuring the α2 chain of the type V collagen C-propeptide (PRO-C5) in serum from patients with cancer. In a discovery cohort we found that serum PRO-C5 was increased in patients with PDAC compared to healthy controls. Furthermore, high levels of PRO-C5 were associated with locally advanced or metastatic PDAC and patients with PDAC with high serum PRO-C5 had increased risk of dying compared to patients with low levels. The results were validated in a large group of 800 patients with PDAC where high PRO-C5 was associated with poor OS in stage II, III and IV independent of other risk factors including PS and CA19-9. Interestingly, the largest relative difference in OS was seen for patients in stage II where the median OS was more than 1 year longer in patients with low PRO-C5 compared to patients with high PRO-C5. In comparison, CA19-9 was also independently associated with OS in patients with stage III and IV disease, but not for patients in stage II. We also showed a relevance of PRO-C5 in other solid tumor types as supported by the gene-expression of the α2 chain that has been shown to be upregulated in many different types of cancer indications (Barsky et al., 1982; Imamura et al., 1995; Sun et al., 2011; Berchtold et al., 2015; Mak et al., 2016; Huang et al., 2017; Ren et al., 2018; Tan and Chen, 2018; Balancin et al., 2020; Wang et al., 2021a; Tan et al., 2021).

Similar to PRO-C5, other biomarkers originating from the tumor fibrotic compartment have shown potential as prognostic and predictive biomarkers in various types of cancers including PDAC (SN et al., 2018; Willumsen et al., 2018; Jensen et al., 2020a; Jensen et al., 2020b; Nissen et al., 2021a; Nissen et al., 2021b; Wang et al., 2021b; Jensen et al., 2021; Nissen et al., 2022; Willumsen et al., 2022). PRO-C3, measuring the formation of type III collagen, has been reported to be prognostic for OS in patients with PDAC (Willumsen et al., 2019; Chen et al., 2020; Nissen et al., 2022). Moreover, the turnover of type III collagen is predictive of response to PEGPH20, an experimental anti-fibrotic drug (Wang et al., 2021b). Like type I collagen, type III collagen is a major fibrillar collagen present in both healthy and diseased tissue (Ricard-Blum, 2011). In contrast to type I and type III collagen which make up the bulk of a fiber, type V collagen is regarded as a minor collagen assisting the major fibrillar collagens in the assembly of a fiber. Lately, other minor collagens, such as the fibrillar type XI and the FACIT collagens (type XIX, XX and XXII) have gained interest as more disease specific biomarkers and/or targets. Similar to what we show for type V collagen here, our group have earlier shown that biomarkers that measure type XI, XIX, XX and XXII collagens are also upregulated in different types of cancers (Thorlacius-Ussing et al., 2020; Nissen et al., 2021a; Madsen et al., 2022; Thorlacius-Ussing et al., 2022). In addition, high serum levels of type XI, XX and XXII collagens were all prognostic for short OS in patients with PDAC (Nissen et al., 2021a; Madsen et al., 2022; Thorlacius-Ussing et al., 2022). These minor collagens are mainly expressed in high levels during embryogenesis and in cancer progression, compared to the relative upregulation of major collagens, which are already present in high amount. Despite the fact that the major type I collagen is important for tumor progression and interacts closely with type V collagen, a biomarker measuring serum type I collagen formation was not increased in the same cohort of different tumor types compared to controls as measured here for PRO-C5 (cohort 3) (Madsen et al., 2022). This supports, that alterations in tumor fibrosis goes beyond type I collagen. Part of this explanation may be that type I collagen is the most abundant protein in the body, and therefore may have less sensitivity as a serological biomarker than the less abundant minor type V collagen, which also underlines the important role of type V collagen in tumor fibrosis. PRO-C5 may have the potential to be used as a treatment guide already in stage II PDAC, and to monitor drug efficacy and detect responders to different treatments. Interestingly, the present study suggests that PRO-C5 may be applicable in many other solid cancer types.

Tumor fibrosis results in stiff tissue, and we and others have earlier shown that CAFs produce more linearized and aligned collagen fibers (Drifka et al., 2015; Drifka et al., 2016a; Bolm et al., 2020; Nissen et al., 2022). Cancer cells are believed to use these linearized collagen fibers to metastasize (Drifka et al., 2016b). When the type V collagen/type I collagen ratio increases, the fibrils become smaller *in vitro* (Adachi and Hayashi, 1986; Birk et al., 1990). What this exactly means for biological function is still unknown. Recently, Chen et al., showed that pancreatic cancer cells produce unique type I collagen homotrimers (α1/α1/α1) in comparison to the type I collagen heterotrimers (α1/α2/α1) produced by fibroblasts (Chen et al., 2022). Moreover, they showed that deletion of type I collagen homotrimers increased survival in a PDAC mouse model and increased T-cell infiltration and efficacy toward anti-PD-1 immunotherapy (Chen et al., 2022). Studies have also shown that type V collagen can exist in both homo- and heterotrimers, e.g., as α1(V)_2_α2(V), α1(V)_3_ or α1(V)α2(V)α3(V) (Chanut-Delalande et al., 2001; Chanut-Delalande et al., 2004; Mak et al., 2016), but the impact of this is yet to be determined. It could be discussed if the homotrimer structure of type I collagen could actually be affected by type V collagen. In line with this, Chen et al., showed that pancreatic cancer cells expressing Col1α1, but not Col1α2, had an elevation of Col5α2 expression (Chen et al., 2022). Altogether these data could lead to the hypothesis, that increased type V collagen expression leads to more linearized type I collagen fibers promoting tumor progression and metastasis.

Some confounders in this study should be mentioned. Selection bias in the BIOPAC cohort may occur, as only patients in good performance for operation or palliative chemotherapy were included. Moreover, several publications have shown that PRO-C5 is associated with liver fibrosis, and it could be speculated if the high PRO-C5 levels are an indirect measure of liver metastases (Vassiliadis et al., 2011; Vassiliadis et al., 2012; Leeming et al., 2015). However, we found that the prognostic value of PRO-C5 was independent of liver metastases which support that the measured PRO-C5 originates from the tumor fibrosis compartment.

In conclusion, the C-propeptide of the minor fibrillar type V collagen is elevated in serum from patients with cancer. In addition, high levels are independently associated with short OS in patients with PDAC, especially patients with stage II disease. This emphasizes the importance and complexity of tumor fibrosis. PRO-C5 may have the potential of guidance in many aspects within drug discovery, patient stratification and drug efficacy.

## Data Availability

The raw data supporting the conclusions of this article will be made available by the authors, without undue reservation.

## Supplementary material

Supplementary Figure S1 PRO-C5 levels in independent stages of varies cancer diseases. I: stage I, II: stage II, III: stage III, IV: stage IV.
