# Supplementary figures and images for: High serum levels of the C-propetide of type V collagen (PRO-C5) are prognostic for short overall survival in patients with pancreatic ductal adenocarcinoma
Source: Front Mol Biosci. 2023 Mar 10;10:1158058. doi: 10.3389/fmolb.2023.1158058 (PMC10036831; doi:10.3389/fmolb.2023.1158058)

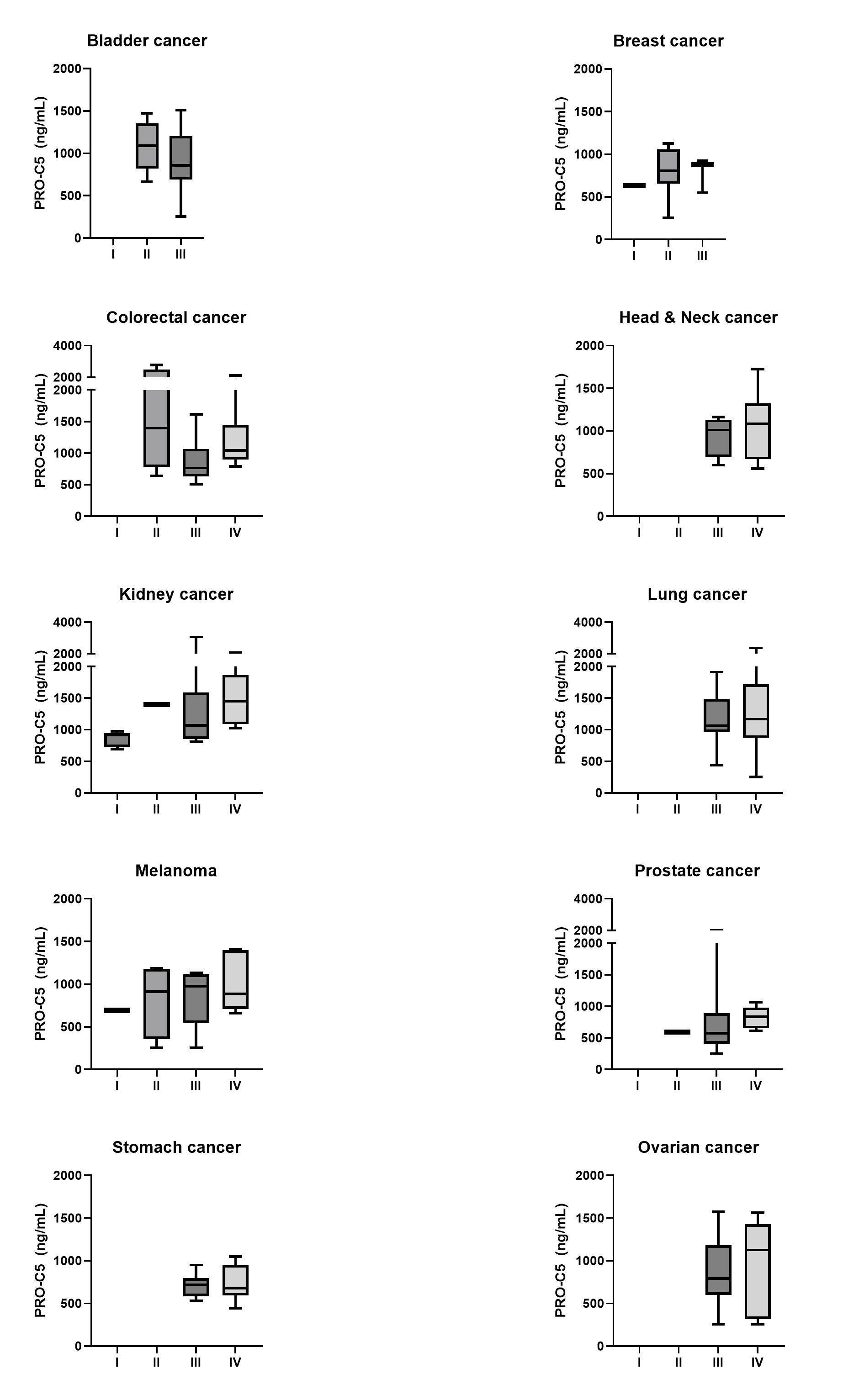

Supplement: Supplementary file 1 [file Image1.JPEG]
